# C-SPAM: an open-source time-resolved specimen vitrification device with light-activated molecules

**DOI:** 10.1107/S2052252523010308

**Published:** 2023-12-14

**Authors:** Alejandra Montaño Romero, Calli Bonin, Edward C. Twomey

**Affiliations:** a Department of Biophysics and Biophysical Chemistry, Johns Hopkins University School of Medicine, Baltimore, MD USA; b The Solomon H. Snyder Department of Neuroscience, Johns Hopkins University School of Medicine, Baltimore MD, USA; c The Beckman Center for Cryo-EM at Johns Hopkins, Johns Hopkins University School of Medicine, Baltimore, MD USA; d Diana Helis Henry Medical Research Foundation, New Orleans, LA USA; Chinese Academy of Sciences, China

**Keywords:** C-SPAM, TR-cryo-EM, sample preparation, photochemistry-coupled devices, millisecond resolution

## Abstract

Molecular structures can be determined *in vitro* and *in situ* with cryo-electron microscopy (cryo-EM). Specimen preparation is a major obstacle in cryo-EM. Typical sample preparation is orders of magnitude slower than biological processes. Time-resolved cryo-EM (TR-cryo-EM) can capture short-lived states. Here, Cryo-EM sample preparation with light-activated molecules (C-SPAM) is presented, an open-source, photochemistry-coupled device for TR-cryo-EM that enables millisecond resolution and tunable timescales across broad biological applications.

## Abstract 2

An open-source platform for light-coupled cryo-electron microscopy specimen preparation is presented.

## 1. Discussion

Fundamental understanding of biological processes requires a description of their structural underpinnings. Through cryo-EM, 3D biological structures *in vitro* and *in situ* can be reconstructed from molecular (∼10 Å) to atomic (∼1.2 Å) resolution (Saibil, 2022 ▸; Lyumkis, 2019 ▸). Samples are prepared for cryo-EM by mixing with a pipette, then depositing the mixture onto a cryo-EM grid. Excess sample or buffer is wicked away by filter paper, and the grid is plunge frozen into a cryogen. This procedure takes seconds to tens of seconds; in most cases, specimen can be prepared on timescales that are orders of magnitude slower than the biological processes of interest; the overall molecular conformations that are fundamental to biology occur on the single millisecond (ms) to 1000 ms timescale. Therefore, trying to correlate biological states observed from functional studies with conformational states reconstructed from cryo-EM is often challenging. Through TR-cryo-EM, non-equilibria states of molecules can be captured by rapidly preparing specimen across a temporal spectrum (Amann *et al.*, 2023 ▸). Generally, the approach of TR-cryo-EM is to activate the system at discreet time points prior to vitrification in the cryogen. Thus, with TR-cryo-EM, molecular conformations can be described as a function of time. The goal of the field has been to capture states on the milliseconds timescale to understand the short-lived states that are fundamental for the non-equilibria of biological processes (Frank, 2017 ▸). Importantly, for drug development, TR-cryo-EM can be utilized with a background of small molecules to describe how small molecules alter molecular conformational ensembles on a physiologically relevant timescale.

There are three principal approaches to TR-cryo-EM: (i) on-grid mixing, (ii) microfluidics and (iii) light-coupled [these techniques are reviewed in great detail by Amann *et al.* (2023 ▸)]. We focus on light-coupled cryo-EM because it is cost effective and adaptable. Light-coupled TR-cryo-EM can be applied to any system where photo-activatable ligands are available (Ménétret *et al.*, 1991 ▸) or to a system that is inherently photo-activatable [*e.g.* bacteriorhodopsin (Subramaniam *et al.*, 1993 ▸)]. Generally, this approach also only requires 2.5 to 3.0 µl of sample deposited on the cryo-EM grid, and thus offers an advantage to microfluidics where larger sample volumes are required to fill capillaries. In addition, the mixing approaches require arrays of different capillary chambers to facilitate different mixing times. Recently, light-coupled TR-cryo-EM devices have been made to analyze ligand-gated ion channels with high temporal resolution (Yoder *et al.*, 2020 ▸; Bhattacharjee *et al.*, 2023 ▸). Though these platforms are fantastic developments, they have two drawbacks. First, the timescales are not readily adaptable; second, single-millisecond resolution, the theoretical limit of TR-cryo-EM (Frank, 2017 ▸; Dubochet, 2007 ▸), is not achievable.

**Figure 1:**
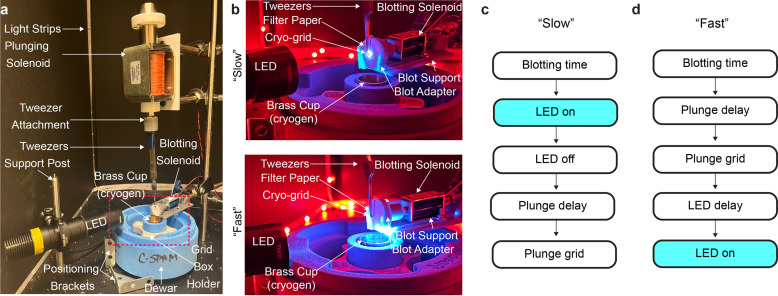
C-SPAM build and operation. (*a*) Overall construction of C-SPAM. All components necessary for operation are labeled. (*b*) Two operations are available with C-SPAM: ‘slow’ and ‘fast’. The slow operation involves the LED at the top position, illuminating the cryo-grid at its starting position. The fast operation involves the LED at the bottom position, illuminating the cryo-grid directly above the brass ethane cup. Order of functions for (*c*) slow and (*d*) fast operations.

**Figure 2:**
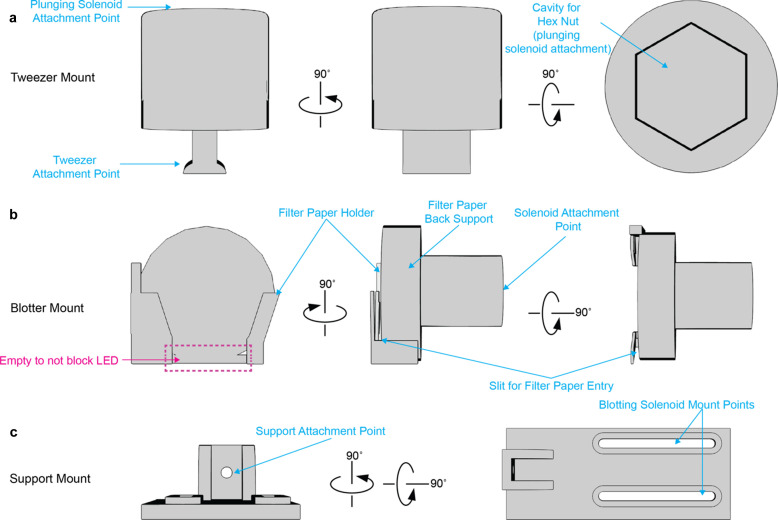
Custom 3D-printed designs for C-SPAM. (*a*) Schematic of the tweezer mount, showing all of the important attachment points. (*b*) Schematic of the blotter mount, showing the placement points for 25 mm filter paper and the solenoid attachment. (*c*) Schematic of the support mount for the blotting solenoid.

Here, we report the development of a new light-coupled TR-cryo-EM platform, cryo-EM sample preparation with light-activated molecules (C-SPAM), which enables users to achieve millisecond-resolution light-coupled TR-cryo-EM and is adaptable to most timescales within single-millisecond increments. Our idea was to construct a system where the light source and cryo-plunger are completely integrated together in a single embedded system. This allows us to control when exactly the light source is coupled to the plunging of the sample. To achieve this, we initially built a light-emitting-diode (LED) into already-existing open-access cryo-EM devices (Rubinstein *et al.*, 2019 ▸; Tan & Rubinstein, 2020 ▸), but designed a new circuit board with an LED driver integrated into it (Fig. S1 of the supporting information). This controls each aspect of C-SPAM [Fig. 1 ▸(*a*)], all of which are either commercially available, have been previously designed and are open source, or are provided here and made open source (Fig. 2 ▸). The main components include: (i) plunging solenoid, (ii) blotting solenoid and (iii) LED [Fig. 1 ▸(*a*)]. The plunging solenoid is connected to cryo-tweezers through a 3D-printed tweezer mount. The blotting solenoid sits on a 3D-printed support mount and is attached to a 3D-printed blotter mount that holds 25 mm filter paper. How all of the components of C-SPAM interact with each other is detailed in Fig. S2 of the supporting information.

**Figure 3:**
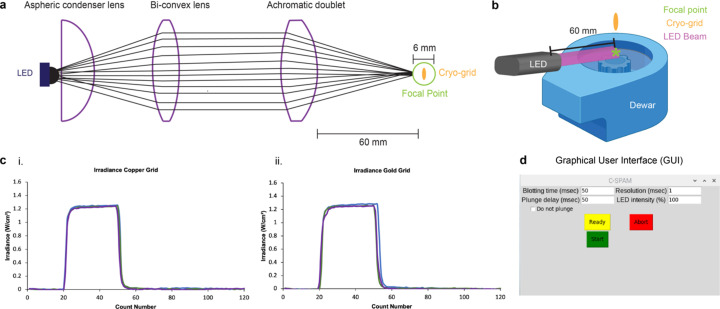
LED lens design, irradiance and GUI. (*a*) Diagram of LED three-lens design, depicting the order and function of each lens. (*b*) Overview of the relationship between the LED focal point and cryo-grid. Dewar in diagram was created with https://www.BioRender.com. (*c*) (i) Irradiance (W cm^−2^) of the LED at 100% with the copper grid present showing a maximum of ∼1.3 W cm^−2^, (ii) irradiance (W cm^−2^) of the LED at 100% with the gold grid present shows the same maximum. Each was run in triplicate. (*d*) C-SPAM GUI showing all available tunable parameters.

We constructed the initial version of C-SPAM with a 365 nm LED because the array of commercially available ligands that are photocleavable at 365 nm makes C-SPAM applicable to many biological questions (Table 1 ▸). This 365 nm LED can be exchanged for other commercially available light sources, making other systems such as optogenetics or thermal sensation available to TR-cryo-EM through C-SPAM (Table 1 ▸). The LED was constructed with a three-lens system, resulting in a 60 mm focal length, which defined where we placed the LED relative to the plunging path [Figs. 3 ▸(*a*) and 3 ▸(*b*)]. The LED is focused to a 6 mm diameter at the focal point, which is roughly twice the diameter of a cryo-grid [Fig. 3 ▸(*a*)]. The focused LED results in an irradiance of ∼1.3 W cm^−2^ at the cryo-grid [Fig. 3 ▸(*c*)], which is sufficient to uncage the commercially available ligands at 365 nm (Bhattacharjee *et al.*, 2023 ▸). For instance, the caged neurotransmitter MNI-glutamate (MNI-Glu) has been successfully used to evoke millisecond currents of glutamate receptors with 50 µs flashes of 7 mW incident light (Matsuzaki *et al.*, 2001 ▸). The LED in our configuration is ∼2.5× stronger than this irradiance. Similarly, MNI-Glu uncaging generated current responses in neurons through photolysis using a 5 mW LED (Passlick & Ellis-Davies, 2018 ▸). In this case, our system is 3.4× stronger than the required irradiance for photolysis of MNI-Glu (Bhattacharjee *et al.*, 2023 ▸). Thus, for this cage, C-SPAM is likely to be efficient. However, the uncaging efficacy will ultimately be caged-ligand-specific.

**Figure 4:**
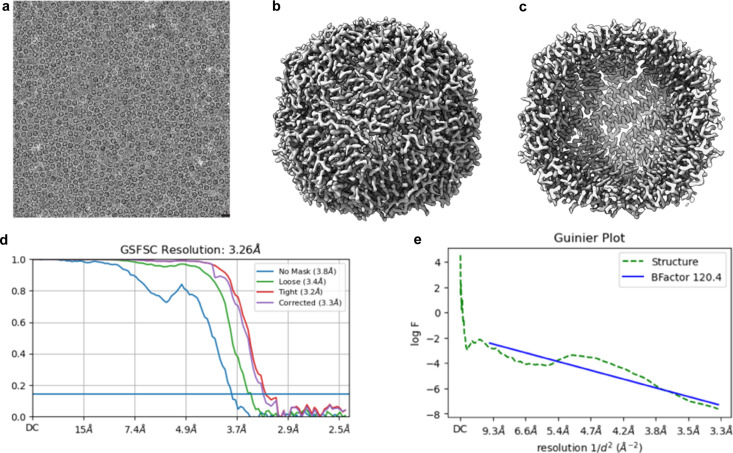
Apoferritin reconstruction using C-SPAM. (*a*) Representative apoferritin micrograph. 20 nm scale bar. (*b*) and (*c*) Reconstruction of apoferritin at 3.26 Å. (*d*) Gold standard Fourier shell correlation (GSFSC) plot from mask auto-tightening in *cryoSPARC*. (*e*) Guinier plot from the reconstruction with the *B*-factor.

Although the heat generated from light activation is a concern, higher powered LEDs used here generate minimal heating and do not affect freezing (Yoder *et al.*, 2020 ▸; Bhattacharjee *et al.*, 2023 ▸). In addition, samples directly heated to 42°C prior to the freezing process do not have a major impact on vitrification (Singh *et al.*, 2019 ▸). Typical 100 nm-thick samples for single-particle cryo-EM vitrify on the sub-millisecond timescale (Dubochet, 2007 ▸; Engstrom *et al.*, 2021 ▸; Kriminski *et al.*, 2003 ▸; Costello, 2006 ▸). A caveat of calculating possible time resolutions with C-SPAM is that the vitrification time will generally vary between specimens and slow the expected temporal resolution. To verify that the system can prepare the sample without irradiation damage, we reconstructed apoferritin with C-SPAM (Fig. 4 ▸).

In the current iteration of C-SPAM there is no enclosure to control humidity and temperature. Thus, specimen preparation is subject to the conditions of the room. For example, in the dark room where we set up C-SPAM, there is an average humidity of ∼35% and temperature range 18–24°C. To verify conditions, we measured the temperature at the cryo-grid and immediately above the liquid ethane cryogen. The cryo-grid, prior to plunging, is at room temperature (*e.g.* 18°C), and the temperature immediately above the cryogen is on average −41°C. We expect this to reduce the efficiency of photolysis.

Temporal constraints in the C-SPAM setup are due to the physical build of the device. There are two positions that the LED can occupy depending on the desired temporal resolution: ‘slow’ and ‘fast’ [Fig. 1 ▸(*b*)]. The construction of the device allows for easy and convenient movement between these two positions by sliding the LED on an optical post. In the slow position, the LED is positioned where it is aligned on the cryo-grid as it rests on the tweezers before plunging, and the LED irradiates the sample for a defined period prior to plunging [Fig. 1 ▸(*c*)]. This enables TR-cryo-EM for time periods of 100 ms or slower. For the fast position, the user slides the LED down the optical post to be flush with the dewar. Thus, the LED irradiates the sample during plunging before it enters the cryogen [Fig. 1 ▸(*d*)]. In this position, temporal resolution of 1–15 ms is achievable.

**Figure 5:**
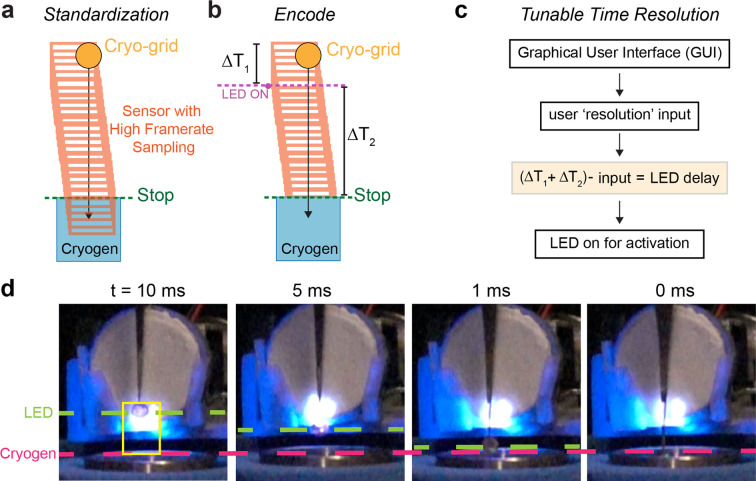
Temporal resolution standardization. (*a*) Standardization of C-SPAM uses a high-frame-rate sampling technique, considering the amount of time the cryo-grid takes to descend into the cryogen from its starting position. (*b*) Encoding of C-SPAM time resolution defines the descending time as (Δ*T*_1_ + Δ*T*_2_), where Δ*T*_1_ is the time between the start of plunging and the switching of the LED on, and Δ*T*_2_ is the time from the start of the reaction to vitrification, representing temporal resolution. (*c*) For tunability of time resolution, an LED delay is encoded in the software that is dependent on both user resolution input and (Δ*T*_1_ + Δ*T*_2_). LED delay is defined as (Δ*T*_1_ + Δ*T*_2_) – input. (*d*) Images of 10, 5, 1 and 0 ms temporal resolutions of C-SPAM.

To reach rapid and adaptable temporal resolutions with C-SPAM, we used a standardization technique that we encoded into the software. For precise standardization, we used a slow-motion camera with a 960 fps setting (∼1.04 ms frame^−1^). The recording started from the movement of the plunging solenoid, through LED powering and ended once the grid was plunged into the ethane cup, representing vitrification [Fig. 5 ▸(*a*)]. By accounting for the number of frames between each point of the process, we determined the time in milliseconds for the grid to descend from its starting point to the liquid ethane. We then defined an LED power delay as the amount of time (Δ*T*_1_) the LED waits to turn on once the plunging solenoid is activated [Fig. 5 ▸(*b*)]. The powering of the LED activates the specimen on the cryo-grid, and the time between this and vitrification in the cryogen (Δ*T*_2_) is the temporal resolution [Fig. 5 ▸(*b*)]. This total value of time is Δ*T*_1_ + Δ*T*_2_. Users of C-SPAM can input which temporal resolution their sample needs to be prepared at, defined as LED delay = (Δ*T*_1_ + Δ*T*_2_) – resolution input [Fig. 5 ▸(*c*)]. In summary, this approach enables the cryo-grid to be irradiated at distinct points prior to vitrification, as the LED is powered at specific points during the plunge based on the power delay and is not continuously powered.

To prepare samples using C-SPAM, the user utilizes the graphical user interface (GUI) that allows for tunable times for blotting, resolution and intensity of the LED [Fig. 3 ▸(*d*)]. Once these parameters are determined for a specific biological system, the user begins by adding sample directly to a grid. As the program starts, the blotting solenoid moves forward to blot the grid, removing excess sample. Once finished, the blotting solenoid retracts and engages the plunging solenoid to begin moving the grid towards the cryogen (Fig. S2 ▸). The LED turns on at a specified point determined by the selected time resolution, activating the reaction [Fig. 5 ▸(*b*)]. For example, we show C-SPAM being utilized for TR-cryo-EM at 10, 5 and 1 ms [Fig. 5 ▸(*c*)]. At each of these time points, through our standardization and encoding of the embedded system, the LED turns on at exactly the user-defined time resolution prior to freezing.

If the user selects the ‘slow’ parameters, the process changes by irradiating the grid for the desired time following blotting, then plunging [Fig. 1 ▸(*c*)]. The C-SPAM software automatically switches to the slow operation if the time resolution entered is ≥50 ms. Because it takes 50 ms for the cryo-grid to descend into the cryogen from the starting position in the current setup of C-SPAM, the minimum time resolution in the slow setting is 100 ms. However, in the fast operation, the cryo-grid can be irradiated at points 15 ms to 1 ms above the cryogen.

There are limitations to this report. For example, to fully validate the C-SPAM device, cryo-EM reconstructions of photoactivated specimen are required. This requires future applications and studies. In addition, future advances in the synthesis of caged molecules will enable a broad spectrum of biological systems to be studied with light-activated TR-cryo-EM approaches such as C-SPAM (Monteiro *et al.*, 2021 ▸). In specialized cases of incomplete photolysis, the concentration of caged molecules in the specimen can be increased to result in suitable concentrations of the released ligand.

C-SPAM is a light-coupled TR-cryo-EM platform that enables investigation of the conformational dynamics of biological molecules at a range of timescales and is adaptable to many areas of biology. In addition, C-SPAM addresses overarching barriers in TR-cryo-EM such as the requirements of financial capital, engineering expertise and high sample volume. All components of this device are available and open source and will thus spur community-driven development of TR-cryo-EM.

## 2. Methods

### 2.1. C-SPAM build

The instrument was built using 3D prints outlined in the Rubenstein device (Rubinstein *et al.*, 2019 ▸; Tan & Rubinstein, 2020 ▸), along with new incorporations and designs as specified. All part names and their availability used in C-SPAM, circuit board schematics and code can be found at https://github.com/twomeylab/c-spam.

The C-SPAM device is built on a breadboard (ThorLabs MB2424) that holds the plunging and blotting solenoids, along with the LED construction. The plunging solenoid (Summit Electronics HD8286-R-F–24VDC) is supported by a 3D-printed holder (Tan & Rubinstein, 2020 ▸) and contains a 3D-printed piece containing magnets (6 × 3MM, purchased from Amazon, secured with ep­oxy) that sits on the solenoid head (Tan & Rubinstein, 2020 ▸). The 3D-printed holder is mounted on a linear motion shaft as described (Tan & Rubinstein, 2020 ▸). This is essential for keeping the solenoid in place while it is de-energized. The plunging solenoid piston contains a 3D-printed spacer (Tan & Rubinstein, 2020 ▸) followed by a 3D-printed tweezer attachment designed in our laboratory (Fig. 2 ▸). The tweezer attachment is compatible with Nanosoft’s cryo-tweezers (Nanosoft 17021002). Furthermore, the base of the blotting solenoid (Digikey 1528–1551-ND) is supported by a 3D-printed support and is connected to a 3D-printed blot adapter, both designed in our laboratory (Fig. 2 ▸). The LED (ThorLabs M365L3) is attached to an optical post (ThorLabs TR6) via a lens tube coupler (ThorLabs SM1TC) and has a three-lens design attached. The optical post is attached to a second optical post through a swivel clamp (ThorLabs SWC/M), which allows the height of the LED to be easily adjusted.

There are three lens tubes that are all coupled. The first lens (ThorLabs ACL25416U-A) is held at the front of the SML10 lens tube, followed by the second lens (Thor Labs LB1596-A) held at the end of the SML03 lens tube, and finishing with the third lens (Thor Labs AC254-050-A-ML) held at the end of the SM1V05 lens tube (Fig. 3 ▸). The LED design results in a 60 mm focal length. To measure irradiance, we used a USB Power Meter Thermal Sensor 0.19–20 µm 10 W Max (ThorLabs PM16-425). All irradiance measurements were taken by placing the power meter 60 mm from the LED build and shining the LED at 100% via the C-SPAM GUI. All readings use a 3 mm diameter and a flat-top beam profile.

The C-SPAM build uses Nanosoft’s vitrification dewar (Nanosoft 21021005) along with its compatible brass ethane cup (Nanosoft 21031002) and grid box holder (Nanosoft 21021002), and is held in place by positioning brackets. All hardware components described are connected to the circuit board (Fig. S1). The circuit board is integrated directly onto the Raspberry Pi 3 Model B+. The full C-SPAM device is powered by a 24 V power supply.

The temperature at the cryo-grid and above the cryogen were measured with a Fluke 51K/J thermocouple.

### 2.2. C-SPAM software

All scripts described are written in Python 3. There are three main scripts that make up the C-SPAM software: (i) *CSPAMgui.py*, (ii) *CSPAMpinlist.py* and (iii) *CSPAMfunctions.py*. The GUI used is encoded by the *CSPAMgui.py* script. The GUI contains four parameters that can be user-defined: (i) blotting time (ms), (ii) plunge delay (ms), (iii) resolution (ms) and (iv) LED intensity (%). In addition, the GUI contains three buttons: (i) Ready, (ii) Start and (iii) Abort (Fig. S2 ▸). The initial GUI was adapted from the Rubinstein device (Tan & Rubinstein, 2020 ▸). The Ready button brings the blotting solenoid forward. The Start button begins the full automated process described. The Abort button stops all processes. The GUI also contains a checkbox of ‘Do Not Plunge’ to impede the plunging solenoid from being energized. Once the user has defined the parameters described, the *CSPAMfunctions.py* script is called to relay the automated process. The hardware components are called by the *CSPAMpinlist.py* script. Though there are two possible time scales, ‘fast’ and ‘slow’, there is only one functions script. In the *CSPAMgui.py* script, we incorporated an ‘if else’ statement that will call either the *applyandplungeFAST* or *applyandplungeSLOW* functions in the *CSPAMfunctions.py* script depending on the user-defined resolution input. If the resolution is ≤15 ms, it will apply the fast function or if it is ≥50 ms, it will apply the slow function.

### 2.3. 3D printing

3D prints were designed in *TinkerCad* (Autodesk, Inc.) and printed with a FormLabs 3+ stereolithography resin 3D printer using FormLabs gray pro resin (FormLabs No. RS-F2-PRGR-01).

### 2.4. Standardization of time resolution

To standardize the time resolution achievable by C-SPAM, we used a high frame-rate sampling technique with a slow-motion camera. With C-SPAM set up in the fast position, we took 960 fps (∼1.04 ms per frame) videos with a Sony RX0 II 1′′ (1.0-type) sensor ultra-compact camera. The videos were analyzed using *Adobe Premiere Pro*. We accounted for the number of frames it took for the grid to reach vitrification from its original position at the start of plunging. This frame number represents the total time the plunging to vitrification process takes, represented by (Δ*T*_1_ + Δ*T*_2_) (Fig. 5 ▸). We used this value to determine the LED delay, defined as the amount of time the LED waits to turn on once the plunging solenoid is activated. We encoded this value as part of the *leddelay* argument in the scripts. To have tunable time resolution with millisecond precision, we made a final LED Delay function that is dependent on both the frame value observed and the user-defined resolution input. The final LED Delay is defined as (Δ*T*_1_ + Δ*T*_2_) – resolution input. Δ*T*_1_ + Δ*T*_2_ is dependent on the physical build of the device. In this first iteration, Δ*T*_1_ + Δ*T*_2_ = 43 ms. Values of both variables change depending on the user input of resolution.

### 2.5. Operation

C-SPAM has two main operations: fast and slow. Both operations begin by the user bringing up the GUI and filling out the parameters. The user then adds 3 µl of sample to the cryo-grid. By pressing the Ready button, the blotting solenoid comes forward to blot excess sample off the grid. If using the fast operation, on pressing Start, the blotting time will begin. Once finished, the blotting solenoid will retract, initiating the plunge delay time. This plunge delay time is essential to prevent crashing of the tweezers and blotting solenoid. Once the delay is finished, this activates the plunging solenoid, in a free-fall mode, to descend towards the cryogen. With the plunging solenoid activated, this calls the LED delay previously described and will turn on the LED at the desired time resolution prior to vitrification. If using the slow operation, the LED will turn on once the blotting has finished. The LED will remain on for the defined resolution time and the plunging solenoid will become activated once the LED turns off. The user can either replace or rotate the 25 mm Whatman paper between each grid.

### 2.6. Cryo-EM

All data were collected on a 200 kV ThermoFisher Glacios equipped with a Falcon 4i camera using ThermoFisher EPU software. For the apoferritin collection, 3 µl of 4 mg ml^−1^ mouse apoferritin was plunge-frozen with C-SPAM in ‘slow’ mode after 4 s blotting with 25 mm Whatman No. 1 filter paper. Micrographs were collected at 1.18 Å per pixel with a 40 e^−^ Å^−2^ dose. All data were imported into *cryoSPARC* (Punjani *et al.*, 2017 ▸) with 40 frames and no upsampling. In total, 154 micrographs were used for processing, blob picking was used to generate templates for picking. Pickled particles were extracted with a 250 pixel box size. A subset of particles was used in *ab initio* reconstruction to generate an initial reference of apoferritin, which was refined to 3.55 Å with a group of 43 620 particles and octahedral symmetry applied. This reconstruction was used as a 3D reference in heterogeneous refinement. In total, 8945 particles from the best class were reconstructed to 3.26 Å with octahedral symmetry.

## 3. Data availability

All 3D-printables, circuit designs, code and scripts designed as part of this study are available at https://github.com/twomeylab/c-spam. All other data and metadata from this study are available from the corresponding author on request.

## Supplementary Material

Supporting figures. DOI: 10.1107/S2052252523010308/fq5023sup1.pdf

## Figures and Tables

**Table:** Commercially available caged molecules that undergo photolysis at 365 nm and are usable with this iteration of C-SPAM (built with 365 nm LED ThorLabs No. M365L3) described in this paper. Demonstrated uses of each molecule can be found at the following references: MNI-glutamate (Canepari *et al.*, 2001 ▸), DPNI-GABA (Trigo *et al.*, 2010 ▸), NPEC-dopamine (Augustin *et al.*, 2018 ▸), NPE-ATP (Zhang *et al.*, 2005 ▸), NPE-proton (Barth & Corrie, 2002 ▸), DMNPE-calcium (Ellis-Davies & Barsotti, 2006 ▸).

Ligands photocleavable at 365 nm		
Molecule	Caged variant	Where to find
Glutamate	MNI-glutamate	Tocris No. 1490
GABA	DPNI-GABA	Tocris No. 2991
Dopamine	NPEC-dopamine	Tocris No. 3992
ATP	NPE-ATP	FisherSci No. A1048
Proton	NPE-proton	Tocris No. 3512
Calcium	DMNPE-calcium	Tocris No. 5948

**Table:** 

Other C-SPAM light sources		
λ (nm)	Format	Where to find
280	LED	ThorLabs No. M280L6
405	LED	ThorLabs No. M405L4
470	LED	ThorLabs No. M470L5
680	LED	ThorLabs No. M680L4
780	LED	ThorLabs No. M780L3
1100	LED	ThorLabs No. M1100L1
1310	Laser	ThorLabs No. S3FC1310
1559	Laser	ThorLabs No. S3FC1550

## References

[bb1] Amann, S. J., Keihsler, D., Bodrug, T., Brown, N. G. & Haselbach, D. (2023). *Structure*, **31**, 4–19. 10.1016/j.str.2022.11.014 PMC9825670 36584678

[bb2] Augustin, S. M., Chancey, J. H. & Lovinger, D. M. (2018). *Cell. Rep.* **24**, 2883–2893. 10.1016/j.celrep.2018.08.042 PMC6182771 30208314

[bb3] Barth, A. & Corrie, J. E. T. (2002). *Biophys. J.* **83**, 2864–2871. 10.1016/S0006-3495(02)75295-8 PMC1302370 12414718

[bb4] Bhattacharjee, B., Rahman, M. M., Hibbs, R. E. & Stowell, M. H. B. (2023). *Front. Mol. Biosci.* **10**, 11129225. 10.3389/fmolb.2023.1129225 PMC10028177 36959978

[bb5] Canepari, M., Nelson, L., Papageorgiou, G., Corrie, J. E. & Ogden, D. (2001). *J. Neurosci. Methods*, **112**, 29–42. 10.1016/s0165-0270(01)00451-4 11640955

[bb6] Costello, M. J. (2006). *Ultrastruct. Pathol.* **30**, 361–371. 10.1080/01913120600932735 17090515

[bb7] Dubochet, J. (2007). *Methods Cell Biol.* **79**, 7–21. 10.1016/S0091-679X(06)79001-X 17327150

[bb8] Ellis-Davies, G. C. R. & Barsotti, R. J. (2006). *Cell Calcium*, **39**, 75–83. 10.1016/j.ceca.2005.10.003 16303177

[bb9] Engstrom, T., Clinger, J. A., Spoth, K. A., Clarke, O. B., Closs, D. S., Jayne, R., Apker, B. A. & Thorne, R. E. (2021). *IUCrJ*, **8**, 867–877. 10.1107/S2052252521008095 PMC8562666 34804541

[bb10] Frank, J. (2017). *J. Struct. Biol.* **200**, 303–306. 10.1016/j.jsb.2017.06.005 PMC5732889 28625887

[bb11] Kriminski, S., Kazmierczak, M. & Thorne, R. E. (2003). *Acta Cryst.* D**59**, 697–708. 10.1107/s0907444903002713 12657789

[bb12] Lyumkis, D. (2019). *J. Biol. Chem.* **294**, 5181–5197. 10.1074/jbc.REV118.005602 PMC6442032 30804214

[bb13] Matsuzaki, M., Ellis-Davies, G. C., Nemoto, T., Miyashita, Y., Iino, M. & Kasai, H. (2001). *Nat. Neurosci.* **4**, 1086–1092. 10.1038/nn736 PMC4229049 11687814

[bb14] Ménétret, J.-F., Hofmann, W., Schröder, R. R., Rapp, G. & Goody, R. S. (1991). *J. Mol. Biol.* **219**, 139–144. 10.1016/0022-2836(91)90554-j 2038049

[bb15] Monteiro, D. C. F., Amoah, E., Rogers, C. & Pearson, A. R. (2021). *Acta Cryst.* D**77**, 1218–1232. 10.1107/S2059798321008809 PMC8489231 34605426

[bb16] Passlick, S. & Ellis-Davies, G. C. R. (2018). *J. Neurosci. Methods*, **293**, 321–328. 10.1016/j.jneumeth.2017.10.013 PMC5705412 29051090

[bb17] Punjani, A., Rubinstein, J. L., Fleet, D. J. & Brubaker, M. A. (2017). *Nat. Methods*, **14**, 290–296. 10.1038/nmeth.4169 28165473

[bb18] Rubinstein, J. L., Guo, H., Ripstein, Z. A., Haydaroglu, A., Au, A., Yip, C. M., Di Trani, J. M., Benlekbir, S. & Kwok, T. (2019). *Acta Cryst.* D**75**, 1063–1070. 10.1107/S2059798319014372 PMC6889916 31793900

[bb19] Saibil, H. R. (2022). *Mol. Cell*, **82**, 274–284. 10.1016/j.molcel.2021.12.016 35063096

[bb20] Singh, A. K., McGoldrick, L. L., Demirkhanyan, L., Leslie, M., Zakharian, E. & Sobolevsky, A. I. (2019). *Nat. Struct. Mol. Biol.* **26**, 994–998. 10.1038/s41594-019-0318-7 PMC6858569 31636415

[bb21] Subramaniam, S., Gerstein, M., Oesterhelt, D. & Henderson, R. (1993). *EMBO J.* **12**, 1–8. 10.1002/j.1460-2075.1993.tb05625.x PMC413169 8428572

[bb22] Tan, Y. Z. & Rubinstein, J. L. (2020). *Acta Cryst.* D**76**, 1092–1103. 10.1107/S2059798320012474 33135680

[bb23] Trigo, F. F., Bouhours, B., Rostaing, P., Papageorgiou, G., Corrie, J. E. T., Triller, A., Ogden, D. & Marty, A. (2010). *Neuron*, **66**, 235–247. 10.1016/j.neuron.2010.03.030 20435000

[bb24] Yoder, N., Jalali-Yazdi, F., Noreng, S., Houser, A., Baconguis, I. & Gouaux, E. (2020). *J. Struct. Biol.* **212**, 107624. 10.1016/j.jsb.2020.107624 PMC7959588 32950604

[bb25] Zhang, L., Buchet, R. & Azzar, G. (2005). *Biochem. Biophys. Res. Commun.* **328**, 591–594. 10.1016/j.bbrc.2005.01.023 15694389

