# Supplementary material for: C-SPAM: an open-source time-resolved specimen vitrification device with light-activated molecules
Source: IUCrJ. 2023 Dec 14;11(Pt 1):16–22. doi: 10.1107/S2052252523010308 (PMC10833387; doi:10.1107/S2052252523010308)
Supplement: Supplementary file 1 [file m-11-00016-sup1.pdf]

IUCrJ

Volume 11 (2024)

Supporting information for article:

C-SPAM: an open-source time-resolved specimen vitrification device with light-activated molecules

Alejandra Montaño-Romero, Calli Bonin and Edward C. Twomey

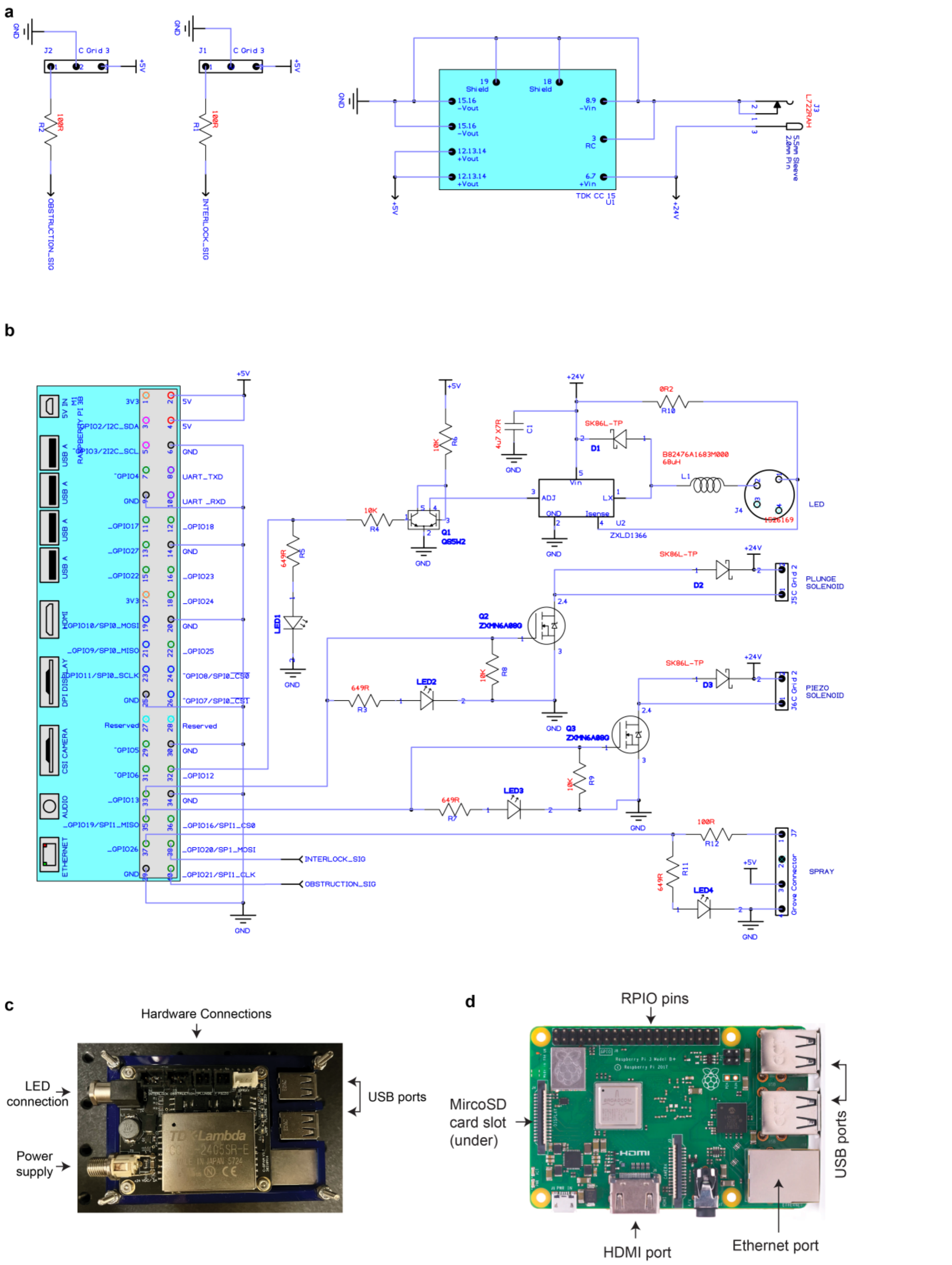

Figure S1 **Circuit schematic and electrical components of C-SPAM. a-b**, Circuit board schematic for incorporation onto Raspberry Pi Model 3 B+. **c**, Fully assembled circuit and Raspberry Pi Model 3 B+. Connections for LED, hardware components, USB ports, and power supply are shown. **d**, Overview of Raspberry Pi Model 3 B+.

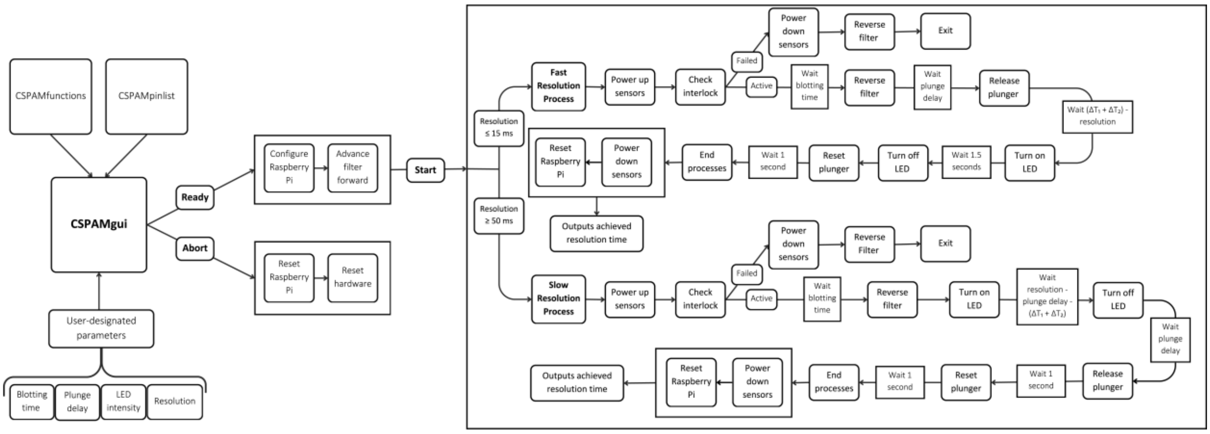

Figure S2 **Software components.** Diagram shows sequence of software operations in C-SPAM components.
